# As coleções coloniais da Universidade de Lisboa: critérios para a caracterização de patrimónios sensíveis

**DOI:** 10.1590/S0104-59702025000100030

**Published:** 2025-09-08

**Authors:** Catarina Simões, Catarina Mateus, Ana Godinho

**Affiliations:** i CHAM - Centro de Humanidades,Faculdade de Ciências Sociais e Humanas/Universidade Nova de Lisboa. Lisboa – Portugal catarinasimoes@fcsh.unl.pt; ii Museu Nacional de História Natural e da Ciência/Universidade de Lisboa. Lisboa – Portugal catarina.mateus@museus.ulisboa.pt; iii Museu Nacional de História Natural e da Ciência/Universidade de Lisboa. Lisboa – Portugal anagodinhocarvalho@museus.ulisboa.pt

**Keywords:** Coleções científicas, Ciência colonial, Coleções sensíveis, Museus e ética, Scientific collections, Colonial science, Sensitive collections, Museums and ethics

## Abstract

As coleções do Instituto de Investigação Científica Tropical foram integradas na Universidade de Lisboa em 2015, ficando o Museu Nacional de História Natural e da Ciência com a responsabilidade pela sua gestão, preservação e acesso. Esse património histórico-científico inclui arquivos, bibliotecas, coleções de história natural, arqueológicas e etnográficas, instrumentos científicos e o Jardim Botânico Tropical. As coleções foram, na sua maioria, constituídas no âmbito de missões científicas coloniais promovidas pelo Estado português nos séculos XIX e XX. Este artigo reflete sobre esse património à luz dos debates contemporâneos sobre os legados do colonialismo, apresentando o trabalho que o Museu tem desenvolvido no tratamento, na identificação e na caracterização das suas coleções histórica e culturalmente sensíveis.

## Abstract (en)

The collections of the Instituto de Investigação Científica Tropical were integrated into the Universidade de Lisboa in 2015, leaving the Museu Nacional de História Natural e da Ciência responsible for their management, preservation and access. This historical and scientific heritage includes archives, libraries, natural history, archaeological and ethnographic collections, scientific instruments and the Jardim Botânico Tropical. Most of the collections were created as part of colonial scientific missions promoted by the Portuguese government during the nineteenth and twentieth centuries. This article reflects on this heritage in light of contemporary debates on the legacies of colonialism, presenting the museum’s work to address, identify and describe its historically and culturally sensitive collections.

Em 2015, o património e as coleções do Instituto de Investigação Científica Tropical (IICT) foram, por decisão do governo português (Portugal, 31 jul. 2015), integrados na Universidade de Lisboa sob gestão do Museu Nacional de História Natural e da Ciência (Muhnac). Essa integração aumentou, de forma significativa, o património do museu, que se tornou o museu português com o mais vasto acervo de coleções científicas coloniais (Casanova, Romeiras, 2020, p.2). Esse património histórico-científico inclui o Jardim Botânico Tropical, em Belém, arquivos, bibliotecas, bem como coleções de fotografia, cartografia, arqueologia, etnografia, antropologia física, geologia, botânica, zoologia e geodesia, num total de três milhões de objetos e cerca de cinco quilómetros lineares de documentação. Embora as coleções contenham exemplares e objetos datados do século XVI ao século XXI, na sua maioria foram constituídas no âmbito de missões científicas oficiais, promovidas pelo Estado português, na sua capacidade de Estado colonizador, entre o último quartel do século XIX e as independências ocorridas em 1975. Neste artigo, analisa-se o trabalho de identificação e caracterização desse património, no âmbito de um programa mais amplo de ressignificação pós-colonial.

Para uma abordagem dessas coleções, é impreterível atentar na história do IICT, instituição herdeira da Junta de Investigações do Ultramar. Esse organismo governamental, que operou sob vários nomes e conheceu diversas reformas e atribuições durante o longo período da sua existência, foi responsável pela concepção e coordenação, a nível central, da política de investigação científica colonial em Portugal (Castelo, 2021a), tendo a sua origem em 1883, com a fundação da Comissão de Cartografia. Integrada no Ministério dos Negócios da Marinha e do Ultramar, a Comissão tinha, inicialmente, como principal desígnio proceder ao levantamento cartográfico dos territórios colonizados pelo Estado português, tendo participado também em negociações diplomáticas com outros países europeus para definição e demarcação de fronteiras em África (Santos, P.C., 2006). Em 1936, num contexto de particular entusiasmo colonial que se verificou em Portugal, com a instauração do Estado Novo,^1^ como também em vários outros países da Europa (Alexandre, 2000), foi criada a Junta das Missões Geográficas e de Investigações Coloniais, afeta ao Ministério das Colónias, para substituir a Comissão de Cartografia e promover a investigação científica nas colónias, alargando os seus horizontes de ação a outros campos do conhecimento. Assim, sobretudo a partir das décadas de 1940 e 1950, e até às independências ocorridas em 1975, essa instituição organizou inúmeras missões científicas em Angola, Moçambique, Cabo Verde, Guiné-Bissau, São Tomé e Príncipe, Timor-Leste, Macau e Índia, com vista a uma verdadeira ocupação científica das colónias (Castelo, 2012b). Foi no âmbito dessas missões científicas oficiais, cujo derradeiro objetivo era o de contribuir para o sucesso da colonização e otimizar a exploração e a rentabilização dos recursos naturais, materiais e humanos dos territórios colonizados, que foram constituídas as coleções do IICT (Godinho et al., 2021). Para além disso, assumindo o Estado Novo o império como verdadeiro desígnio e imperativo nacional, e instrumentalizando-0, estruturalmente, como uma componente fundamental da sua propaganda nacionalista (Rosas, 1995; Jerónimo, 2010), o conhecimento que era produzido sobre as colónias era mobilizado para exposições e programas comemorativos destinados a projetar o país nacional e internacionalmente, sendo, assim, fundamental para os grandes momentos de propaganda do regime, de que são exemplos por excelência a Exposição Colonial Portuguesa de 1934 ou a Exposição do Mundo Português de 1940 (Acciaiuoli, 1998).

Com o golpe militar de 25 de abril de 1974, que colocou um fim à ditadura, reconheceramse as independências de Guiné-Bissau (1974), Moçambique, São Tomé e Príncipe, Angola e Timor-Leste (1975), e assistiu-se a uma longa, mas tímida, reestruturação da Junta de Investigações Científicas do Ultramar.^2^ Em 1979, a Junta tornou-se Laboratório Nacional de Investigação Científica Tropical e, em 1982, assumiu a sua designação final de Instituto de Investigação Científica Tropical (IICT). Pretendeu-se, com essa reforma, transformar a instituição anteriormente responsável pela produção de conhecimento científico sobre as colónias num instituto vocacionado para a cooperação científica e técnica com os países recém-independentes (Lobato, 1983). Em resultado, nas últimas décadas do século XX, o IICT assumiu como um dos seus principais objetivos a divulgação e disponibilização das suas coleções científicas aos países membros da Comunidade dos Países de Língua Portuguesa. Esse ideal de cooperação foi acompanhado por um esforço evidente da instituição no sentido de construir e promover uma narrativa positiva, por vezes até autocelebratória, sobre a sua história e o seu legado, no âmbito da ciência “tropical”, com uma sucessão de exposições e programas comemorativos organizados já nas primeiras décadas do século XXI (Castelo, 2012a; Nunes, Roque, 2008).

O processo de incorporação do IICT na Universidade de Lisboa, em 2015, introduziu de forma clara uma nova etapa na vida da instituição e das suas coleções. Desde logo, ao passar a ser gerido por um museu, o património foi naturalmente revestido de uma lógica de divulgação pública bastante mais acentuada, nomeadamente do ponto de vista de exposições e programação cultural. Por outro lado, o património integra uma universidade, onde se espera que a problematização e o debate sejam centrais. Finalmente, a transição ocorreu numa altura de intensa visibilidade, sobretudo a nível internacional, dos debates em torno dos patrimónios coloniais e da necessidade de se reconsiderarem as narrativas dominantes sobre os legados dos impérios europeus. Foram, nesse contexto, marcos especialmente relevantes, entre outros: a publicação do relatório elaborado por Felwine Sarr e Bénédicte Savoy, por encomenda do presidente francês Emmanuel Macron, sobre o património cultural africano em coleções públicas francesas, incluindo recomendações sobre restituições (Sarr, Savoy, 2018); o lançamento de um guia prático, pela Associação de Museus Alemães, contendo orientações sobre coleções provenientes de contextos coloniais (DMB, 2021);^3^ e a publicação, por parte do Tropenmuseum de Amsterdão, de uma lista de palavras consideradas problemáticas e de termos alternativos que devem ser utilizados pelos museus e outras instituições culturais, partindo do pressuposto de que a linguagem utilizada pode condicionar o sentimento de pertença de certos grupos, fazendo-os sentirem-se mais ou menos marginalizados ou integrados na sociedade (Modest, Lelijveld, 2018).

A integração do património do IICT no Muhnac tem, desse modo, vindo a beneficiar de uma extensa produção intelectual sobre o colonialismo e a memória dos impérios. O processo tem sido influenciado, também, por significativas alterações de fundo que se verificaram, nos últimos anos, na forma como os museus do Norte Global estudam, gerem e comunicam as suas coleções coloniais (Pabst, 2019; Message, 2019; Brandstetter, Hierholzer, 2018).

Esse contexto foi favorável a que, pela primeira vez, esse património, e em particular os seus contextos de recolha, fossem encarados, a um nível institucional, com uma visão crítica, conduzindo a um reconhecimento de que essas são coleções sensíveis, com uma história problemática que deve ser apresentada de forma rigorosa, explícita e transparente. Por outro lado, motivou um questionamento estrutural e alargado sobre as formas como essas coleções devem, no presente, ser mobilizadas, estudadas e apresentadas, procurando implementar-se as atuais recomendações internacionais no que diz respeito ao tratamento de coleções coloniais.

Sendo consensual o reconhecimento de que as instituições museológicas têm o dever ético e legal de promover investigações cuidadas sobre a proveniência dos objetos que albergam, e que esta deve mesmo ser uma das funções essenciais dos museus (DMB, 2021, p.10), desde 2018 está em curso no Muhnac, de forma continuada, o aprofundamento do estudo sobre os contextos de constituição das coleções associadas às missões científicas coloniais a Angola, Moçambique, Guiné-Bissau e Timor-Leste. É importante notar, a esse respeito, que, dado o carácter institucional dessas missões, esta pesquisa tem sido consideravelmente facilitada pela elevada taxa de inventariação prévia dos objetos, bem como pela profusa documentação primária que se lhes encontra associada, e que inclui cadernos de campo, relatórios, documentação administrativa e fotografias. Nessa documentação encontram-se dados fundamentais que contextualizam os materiais, objetos e espécimes, a forma como foram recolhidos ou adquiridos e constitui um repositório de informação crucial para a compreensão das coleções.

O estudo e a caracterização dessas coleções beneficiaram igualmente da existência de uma vasta produção académica, principalmente do âmbito da história e das ciências sociais, sobre algumas dessas coleções ou de seus elementos específicos, bem como sobre a história do IICT e a política científica colonial portuguesa do século XX. Uma parte significativa do trabalho de análise e reinterpretação das coleções aqui apresentado tem sido feita com base em literatura produzida por diversos investigadores externos à equipa do Muhnac. Alguns desses trabalhos foram produzidos por investigadores que, em determinadas fases dos seus percursos académicos e profissionais, tiveram uma afiliação institucional ao IICT; outros são de autores que nunca tiveram qualquer associação a essa instituição. Uns de caráter mais descritivo, outros mais analíticos, todos esses contributos têm se revelado fundamentais para o aprofundamento do conhecimento por parte dos técnicos e curadores do Museu acerca dessas coleções e para a melhor compreensão sobre os seus contextos de constituição. Isso evidencia a importância do trabalho de investigação independente e rigoroso, produzido no contexto da academia, para a atualização dos discursos e narrativas que se constroem e disseminam, e também para a adoção de perspetivas e atitudes mais críticas e eticamente comprometidas por parte das instituições museológicas que têm à sua guarda patrimónios difíceis e que contam, não raras vezes, com recursos humanos limitados e falta de investigadores especializados entre os seus quadros.

É importante referir, nessa sequência, que, para além das coleções do IICT, o museu, que teve a sua origem no Real Museu de História Natural e Jardim Botânico da Ajuda, criado em 1768, tem outras coleções coloniais – nomeadamente coleções geológicas, biológicas e documentais que testemunham as apropriações científicas e políticas da natureza de territórios ocupados pelo Estado português entre os séculos XVIII e XX, e que foram objeto de uma recente panorâmica (Póvoas, Correia, Alves, 2021). Entre essas coleções contam-se, no arquivo histórico do Museu, listagens de remessas de materiais geológicos, botânicos e zoológicos, sobretudo provenientes do Brasil, mas também de Cabo Verde, Moçambique e da Índia, bem como relatos e descrições desses territórios, e um extenso acervo de desenhos científicos, enviados sobretudo por administradores coloniais, militares, naturalistas ao serviço do reino e particulares desde o final do século XVIII e ao longo do século XIX; exemplares botânicos e geológicos do mesmo período e das mesmas proveniências geográficas; e coleções geológicas, mineralógicas, paleontológicas e de estratigrafia (e respetiva documentação associada), constituídas sobretudo no âmbito da missões geológicas e geográficas oficiais e outras expedições a Angola, Cabo Verde, Moçambique, São Tomé e Príncipe e Timor-Leste durante o século XX (Póvoas, Correia, Alves, 2021).

A história do Muhnac, das suas coleções, e da sua relação com a produção de conhecimento científico, em Portugal, sobre a natureza dos territórios colonizados tem sido objeto de abundante produção historiográfica recente (vejam-se, entre outros, Póvoas et al., 2011; Ceríaco, 2014; Felismino, 2015; Brandão, Póvoas, Lopes, 2015; Madruga, 2019; Albuquerque, Figueiróa, Felismino, 2020).

Os critérios adotados para a caracterização das coleções e a identificação de objetos sensíveis baseou-se, sobretudo, no já mencionado manual de boas práticas da Associação dos Museus Alemães. Segundo esse documento, o reconhecimento do caráter sensível das coleções provenientes de contextos coloniais é essencial para a sua análise crítica. Por outro lado, é igualmente fundamental destrinçar os diferentes níveis de sensibilidade associados a diferentes tipologias de objetos, de forma a adequar as práticas e os discursos relativos a eles. Segundo esse guia, deve considerar-se como “historicamente sensível” qualquer objeto adquirido ou produzido em contexto colonial, na medida em que a sua aquisição, utilização ou produção envolveu frequentemente o uso da força, promovendo ou reforçando o estabelecimento de relações de domínio e dependência. Para além disso, muitos desses objetos refletem e veiculam ideias coloniais, racistas ou discriminatórias, devendo, por isso, a sua história e natureza ser analisada e transmitida de forma cuidada pelos museus. Também necessária é a identificação de objetos “culturalmente sensíveis”, categoria que diz respeito não ao contexto de produção e/ou aquisição dos objetos, mas ao significado que possam ter para as respetivas comunidades de origem; objetos cerimoniais ou religiosos, bem como quaisquer tipos de restos humanos, e por vezes também imagens de pessoas registadas em fotografia ou vídeo, enquadram-se nessa categoria de materiais “culturalmente sensíveis”, devendo ser considerados especialmente problemáticos. Com efeito, como é explicado em *Guidelines for German museums care of collections from colonial contexts* (DMB, 2021), com frequência esses tipos de objetos são sujeitos a normas específicas de manuseamento ou a restrições de acesso no contexto das suas comunidades de origem, podendo mesmo ser considerados perigosos, sendo imprescindível que se faça a sua identificação e que se estabeleçam procedimentos adequados para o seu tratamento, a sua descrição e a sua disponibilização aos públicos, idealmente, num processo colaborativo e de envolvimento ativo com as comunidades cujos membros se consideram descendentes dos indivíduos que, originalmente, produziram ou utilizaram estes objetos (DMB, 2021, p.18-22).

Em suma, o Muhnac assumiu a totalidade das suas coleções coloniais como historicamente sensível, de acordo com as *Guidelines for German museums care of collections from colonial contexts*, e considerou os seguintes critérios para a identificação de objetos como “problemáticos” ou “extremamente” sensíveis: (a) objetos sem documentação de entrada; (b) material biológico humano; (c) representações humanas sensíveis (mulheres, crianças, violência, representações claramente racistas e discriminatórias); (d) outros objetos culturalmente sensíveis; (e) recolhas após as convenções da Organização das Nações Unidas para a Educação, a Ciência e a Cultura (Unesco) de 1970 e 1972); (f) natureza dos agentes de recolha, especialmente militares durante as guerras de resistência contra a ocupação colonial; (g) pertinência científica na época da recolha.^4^ Esses critérios serão detalhados adiante.

Dado o enorme volume de objetos e a heterogeneidade das coleções, a aplicação desses critérios foi feita em duas fases. Na primeira, excluíram-se as coleções de história natural e analisaram-se as coleções com maior potencial de possuírem objetos problemáticos ou extremamente sensíveis: as coleções de etnografia, arqueologia, fotografia e de antropologia física. Ainda que se reconheça o carácter sensível das restantes coleções coloniais do museu – nomeadamente as que resultaram de expedições científicas dos séculos XVIII e XIX ao Brasil e a África, e ainda as coleções de história natural do IICT –, o fato de se ter assumido como prioritário o estudo das coleções com objetos extremamente sensíveis conduziu a que aquelas fossem excluídas dessa primeira fase do trabalho de análise. Essa primeira abordagem permitiu, em setembro de 2021, identificar de forma sistemática o grupo de objetos que serão apresentados adiante. São objetos que têm vindo a ser alvo de estudos mais aprofundados, nomeadamente teses e projetos de investigação. Para além disso, foram redefinidas para cada um as condições de acesso, acondicionamento e exposição. Neste artigo apresentam-se, sobretudo, os resultados dessa primeira fase de identificação, o que justifica o enfoque nas coleções do IICT.

Simultaneamente, avançou-se na segunda fase de identificação e quantificação de objetos histórica e culturalmente sensíveis. A partir de 2022, a análise foi alargada a todas as coleções do museu, de todas as áreas científicas. Para essa análise, que alarga e aprofunda os critérios acima definidos, foram criados dois instrumentos internos de trabalho, nomeadamente uma *checklist* e uma base de dados. Esse trabalho ainda se encontra em curso, e, pela sua natureza e pela vastidão das coleções, é uma tarefa de muito longa duração. Desse modo, o aprofundamento do estudo e da problematização das coleções científicas coloniais cronologicamente mais recuadas, também elas importantes testemunhos do passado imperial português, está em curso, esperando-se que venha a ser apresentado de forma detalhada em breve, num artigo que se encontra em preparação.

O trabalho de identificação levado a cabo e apresentado neste artigo tem servido, desse modo, não só para aferir quais coleções ou objetos devem ser tratados com especial cuidado, mas também para promover uma caracterização sistemática de todas as coleções.

## Critérios: condições de aplicabilidade

Uma das questões mais importantes que se procurou aferir diz respeito aos vários contextos de recolha ou de constituição das coleções, partindo do princípio de que existiram diferentes tipos de contextos coloniais (DMB, 2021, p.30-43). As coleções do IICT foram, na sua maioria, recolhidas, utilizadas ou produzidas em territórios sob domínio colonial formal; no entanto, existem objetos provenientes de zonas de conflito armado, que na maioria dos territórios terminou após as independências, em 1975, mas que, por exemplo, no caso de Angola, se estendeu, de forma intermitente, até 2002; e há também um conjunto assinalável de objetos de propaganda, que veiculam uma ideologia colonial, como é o caso do conjunto de 14 bustos que se encontram no Jardim Botânico Tropical, em Belém. Essas peças foram produzidas para a Exposição do Mundo Português de 1940, cuja seção colonial se localizava nesse jardim, e que foi um dos mais emblemáticos e representativos atos de propaganda colonial do Estado Novo, a par da Exposição Colonial do Porto, realizada em 1934 (Rodrigues, 2016).

Outro critério que se considerou relevante para essa caracterização das coleções diz respeito aos seus agentes de recolha, entre os quais se contam administradores coloniais, militares, investigadores em missões científicas, mas também comunidades locais, missionários, particulares, entre outros. Tratando-se de coleções científicas, os investigadores das missões de estudo contam-se entre os principais e mais frequentes atores associados à recolha de objetos; contudo, procurou apurar-se os casos em que objetos ou grupos de objetos foram recolhidos por administradores coloniais, e sobretudo por militares, nomeadamente no contexto de guerras de resistência contra o poder colonial,^5^ e no contexto das Guerras de Libertação, por se considerar que esses casos merecem uma atenção particular.

Sobre o papel dos investigadores como agentes de recolha, é importante ressalvar que a produção de ciência e de conhecimento tende frequentemente a ser encarada como uma atividade positiva e desejável, à luz de uma visão que privilegia e legitima noções eurocêntricas de progresso e desenvolvimento, o que facilitou que, durante décadas, se ignorasse o legado dessas missões na sua dimensão profundamente colonial, e tantas vezes violenta e repressiva. Contudo, um olhar atento sobre a documentação associada às missões científicas, nomeadamente fotografias, relatórios e publicações, permite encarar essas coleções como testemunhos reveladores da forma como a ciência colonial era conduzida e das suas agendas políticas. Possibilita, designadamente, contextualizar o trabalho de campo como palco de práticas extrativistas e de relações de poder desiguais, onde a apropriação de territórios colonizados e a exploração dos seus recursos e das suas populações se materializava (Dubald, Madruga, 2022).

As coleções de fotografia,^6^ por exemplo, que documentam detalhada e profusamente as missões científicas, abarcando um período cronológico de quase um século (Godinho et al., 2021), demonstram como a produção de ciência colonial dependia, largamente, da exploração da força de trabalho das populações locais. Tal revela cabalmente que, mesmo em períodos posteriores à abolição da escravatura, o trabalho forçado permaneceu uma realidade normalizada, consagrada e mesmo promovida pelo Estatuto e respetivo Código de Trabalho atribuído às populações africanas colonizadas (Meneses, 2010; Neto, 2010; Jerónimo, 2010).^7^ Era uma prática perfeitamente assimilada e visível nos contextos onde a ciência era produzida, *in situ*, e, por inerência, onde as coleções ganhavam existência (Dubald, Madruga, 2022, p.2). As fotografias documentam os trabalhos de campo e a presença ubíqua de centenas de trabalhadores africanos, a quem eram atribuídos os trabalhos pesados. Simultaneamente, revelam também que esses trabalhadores participavam e contribuíam, de forma ativa e significativa, para a produção de conhecimento, nomeadamente como informadores, intérpretes, guias, doadores, preparadores, construtores e assistentes de trabalho técnico, conforme tem sido demonstrado por ampla literatura sobre o tema (veja-se, a esse propósito, Santos, M.E., 2006; Lobato, 2006; Kennedy, 2013; Raj, 2016). E, embora as legendas dessas imagens destaquem, geralmente, apenas dados técnicos dos trabalhos, os relatórios que as acompanham dão conta dessas diversas formas em que se traduzia a participação de elementos das populações locais nas missões, e revelam as tensões, negociações e convergências entre as agendas coloniais e as práticas e interesses locais. Tem sido, por exemplo, objeto de estudos recentes o importante papel dos médicos tradicionais africanos na transmissão de conhecimento aos cientistas europeus sobre plantas com propriedades terapêuticas (Conde et al., 2014; Marandino, Meneses, 2024). Por outro lado, fontes da história oral revelam a multiplicidade de interações e relações de cooperação que se estabeleciam, de parte a parte, entre cientistas e populações locais, que não se esgotavam nas questões puramente relacionadas com o trabalho de campo, e que ultrapassavam as simples dinâmicas de poder entre colonizadores e colonizados (Castelo, 2020, p.8).

Desse modo, essas coleções e a sua documentação associada testemunham que, embora a história da ciência colonial tenda a focar-se exclusivamente nos contributos dos investigadores e chefes de missões, a sua produção contou com a participação de uma grande diversidade de atores cujas agências e contingências sociais, económicas e políticas raramente são (re) conhecidas. É, ainda assim, importante ressalvar que o envolvimento dos investigadores no projeto colonial – do qual beneficiavam e para o qual contribuíam – nem sempre “se processou sem tensões, negociações e contradições”, existindo casos documentados de divergência e crítica aberta por parte de cientistas, nomeadamente de cientistas sociais, geógrafos e agrónomos, a determinadas opções políticas do Estado colonizador, ainda que esses momentos de contenda não chegassem, na maioria das vezes, a constituir uma verdadeira interrogação sobre a legitimidade do poder e do domínio colonial (Castelo, 2021a, p.478-481, 2020), que continuou, até praticamente à década de 1960, a reunir um consenso geral na sociedade portuguesa (Rosas, 1995).

A coleção de fotografia contém ainda imagens abertamente discriminatórias, que refletem e testemunham modos de pensar claramente racistas. Em particular, as fotografias associadas às coleções de antropobiologia e a documentação científica publicada relacionada, que veiculam uma ideologia colonial baseada na diferenciação racial, onde se construía um discurso sobre a origem da humanidade, por meio do registo de características físicas e da demonstração “científica”, empírica, das diferenças entre seres humanos. Com efeito, uma parte significativa dos estudos conduzidos pelas chamadas missões antropológicas, que estiveram na origem das coleções arqueológicas e etnográficas, tinham como principal desígnio aferir as “características somáticas e possibilidades psicofísicas dos diferentes povos e tribos coloniais”, assumidamente com o objetivo de identificação dos indivíduos mais e menos aptos para o trabalho, numa lógica de rentabilização dos recursos humanos das colónias que igualava a rentabilização dos recursos naturais. Outro dos seus grandes propósitos era, naturalmente, coligir informações com vista a otimizar a administração das populações colonizadas (Castelo, 2012b), tornando assim óbvio o papel da ciência como instrumento ao serviço da agenda política do colonialismo português.

Outro critério relevante tido em consideração na caracterização geral preliminar dessas coleções diz respeito à própria forma de recolha e de incorporação dos objetos, ainda que seja de ressalvar que, nesses contextos, raramente as circunstâncias em que essa recolha ocorreu se consigam apurar com clareza. Será seguro afirmar que terão, certamente, existido nessas missões muitas mais recolhas forçadas do que aquelas que surgem declaradas e discriminadas em relatórios e outra documentação escrita associada às coleções. Existem, ainda assim, alguns exemplos documentados de objetos que foram, assumidamente, segundo as próprias fontes oficiais, apreendidos com o uso da força e sem o consentimento dos seus anteriores proprietários ou tutores, e cujas histórias serão adiante particularizadas de forma mais aprofundada.

Refira-se, ainda, que uma das questões que, estruturalmente, têm ocupado a equipa do Museu é a averiguação sobre a existência de objetos que tenham sido incorporados após as convenções da Unesco de 1970 e 1972, que regulamentaram, respetivamente, a proibição e o impedimento de importação, exportação e transferência ilícita da propriedade de bens culturais, e a proteção dos patrimónios mundial, cultural e natural, e que constituem, ainda hoje, as principais diretivas internacionais para a salvaguarda e preservação do património em escala global.

Por fim, é ainda importante salientar que, tal como temos vindo a referir, dada a natureza oficial das missões científicas no âmbito das quais a maioria desses objetos foram adquiridos, a maioria das coleções encontra-se rigorosa e profusamente documentada. Ainda assim, um critério que foi considerado incontornável foi a identificação de objetos e coleções sem documentação associada e sem informações quanto aos seus contextos de recolha, na medida em que essas exceções configuram casos particularmente problemáticos, em que o estudo de proveniência aprofundado das coleções se torna um exercício praticamente impossível e a sua gestão muito mais desafiante.

## Resultados: os objetos “extremamente” sensíveis do Muhnac

A caracterização das coleções com base nos critérios até aqui enunciados e explicados permitiu a identificação de um conjunto de objetos que foram considerados extremamente sensíveis, tendo em consideração os seus contextos de recolha ou a sua natureza. Como já referimos, as coleções de antropobiologia em geral, que contêm objetos e materiais recolhidos no contexto de missões de antropologia, incluindo materiais biológicos humanos e dados antropométricos, contam-se entre aquelas que são eticamente mais problemáticas, na medida em são exemplos evidentes de uma ciência colonial baseada em pressupostos raciais. O próprio conceito de “antropobiologia”, veiculado por António Mendes Correia, presidente da Junta das Missões Geográficas e de Investigações Coloniais/Junta de Investigações do Ultramar entre 1946 e 1959, diz respeito a uma forma de ciência racial, que cruzava fundamentos da antropometria e da taxonomia racial, adotando técnicas e metodologias que já na década de 1950 se consideravam, internacionalmente, obsoletas e antiquadas (Roque, 2019). O objetivo assumido e oficial dessas missões era, como se pode verificar pela consulta aos decretos-lei da época (Portugal, 3 abr. 1945, 19 jun. 1945), “o estudo da robustez e vitalidade dos indígenas e dos vários grupos étnicos da colónia …; os estudos psicotécnicos experimentais com o objetivo de se colherem elementos que permitam conhecer-se as aptidões dos indígenas para os vários misteres” (Portugal, 19 jun. 1945, p.530). As coleções de antropobiologia incluem, desse modo, registos de medições antropométricas, amostras de sangue, de cabelo e pelos púbicos, impressões digitais e palmares, e retratos de frente e de perfil de milhares de pessoas estudadas e categorizadas em Angola, Guiné-Bissau, Moçambique, Macau, São Tomé e Príncipe e Timor-Leste, sendo que, neste último território, esse tipo de estudo continuou a ser produzido até à década de 1970.

As 59 características físicas recolhidas em fichas somáticas (cor da pele, cabelo, forma das orelhas, do nariz e dos lábios, espessura da massa adiposa, massa muscular, dimensões do tronco, membros e crânio etc.) foram usadas como dados científicos pela chamada “antropologia física”, produzindo e reforçando estereótipos raciais baseados nas características físicas das populações colonizadas. Outros, como as tatuagens e as alterações dentárias intencionais, consideradas como um desvio psicológico, eram também avaliados (Roque, 2006; Cascais, Costa, 2019; Costa, 2022). Para além das fichas somáticas, cada indivíduo era também fotografado de frente e de perfil, e as suas impressões digitais registadas, seguindo a técnica *bertillonage*.^8^ Esses materiais testemunham o domínio e a subjugação exercidos pela ciência colonial e os seus agentes, sendo referido nos relatórios produzidos e nos artigos publicados que era prática habitual recorrer-se à coação e a pagamentos. Percebe-se, também, pelas descrições, que as condições em que eram realizadas as observações médicas não garantiam – nem tinham, aliás, como preocupação garantir –a segurança e a dignidade das pessoas avaliadas (Correa, 2022). Fotografias e filmes, utilizados como ferramenta essencial para esses registos, integram hoje essas coleções, num vasto manancial de dados recolhidos com vista à produção de uma ciência que era encarada, pelo regime colonial, como uma via para a construção de uma comunidade multirracial que prometia eternizar a prosperidade e a glória imperial do Estado português. Uma ideia que era vital para um regime cujo nacionalismo se baseava, largamente, na nostalgia dos feitos dos chamados “Descobrimentos” (Roque, 2019).

**Figura 2:**
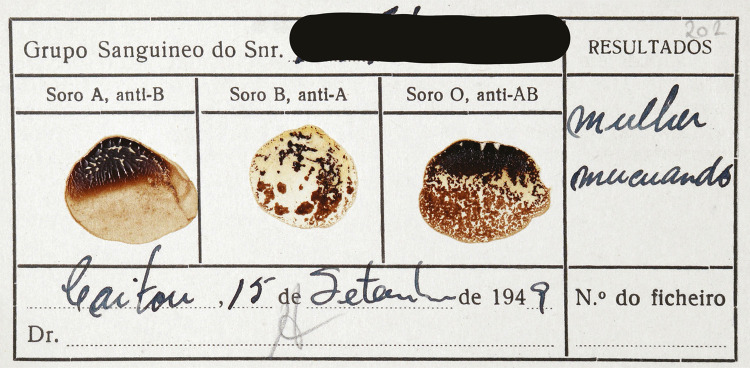
: Cartão com amostra de sangue, Missão Antropológica de Angola, campanha de 1949. Nome da pessoa rasurado pelas autoras (Cartão..., 1949)

Sendo a totalidade das coleções de antropobiologia extremamente sensível, três grupos de objetos são de particularizar pela sua natureza especialmente problemática. O primeiro, um conjunto de esqueletos provenientes de Moçambique, alguns dos quais com dados pessoais associados. O segundo, um conjunto de 16 mil cartões com amostras secas de sangue e grupos sanguíneos humanos originários de populações africanas e asiáticas, recolhidos no contexto de expedições da década de 1950, e recentemente estudados e problematizados (Roque, 2019). Também alguns desses cartões contêm dados pessoais associados, nomeadamente os nomes das pessoas de quem as amostras haviam sido recolhidas (Figura 2). Para além disso, é ainda de referir um grupo de fotografias da Missão Antropobiológica de Angola, de nudez explícita de mulheres, homens, jovens rapazes e meninos para estudar possíveis casos de macronínfia (hipertrofia dos pequenos lábios da vulva), esteatopigia (hipertrofia das nádegas devido à acumulação excessiva de gordura) e de posição peniana, abordados como patologias degenerativas pela antropobiologia portuguesa, que se considerava poderem comprometer ou serem transmissíveis aos colonizadores (Cascais, Costa, 2019). Essas fotografias, juntamente com os textos produzidos, testemunham, de forma evidente, não só os estigmas imputados aos corpos colonizados, racializados, no quadro do imaginário colonial (Vicente, 2017; Correa, 2022), mas demonstram também a violência ginecológica à qual as mulheres colonizadas foram submetidas, “com o objetivo de provar uma sexualidade patológica ou hipersexualidade..., ditando a sua perigosidade ou a necessidade da sua ‘domesticação’” (Costa, 2022, p.252).

**Figura 3:**
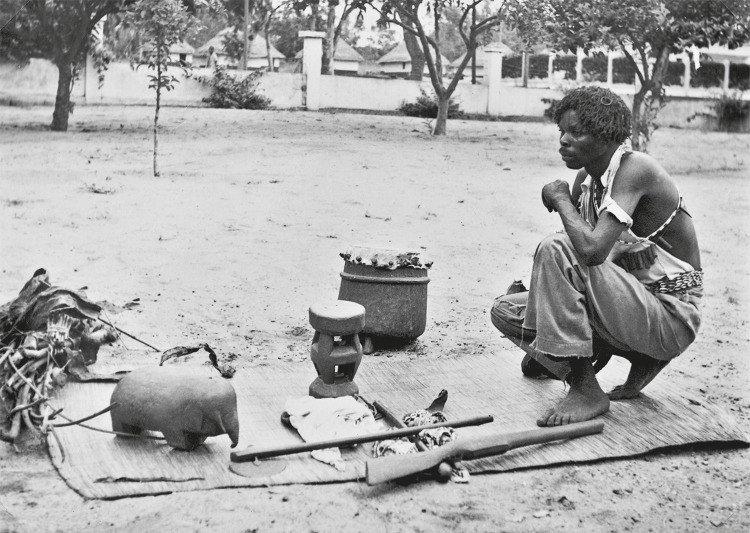
: Artur Murino Mafumo, médico tradicional de Moçambique, com os seus objetos de trabalho posteriormente apreendidos pelas autoridades coloniais, estudados e trazidos pelos investigadores da Missão Antropológica de Moçambique para Portugal, campanha de 1955 (Artur..., 1955)

Foram, igualmente, qualificados como extremamente sensíveis dois grupos de objetos da coleção etnográfica, em que se cruzam e sobrepõem várias categorias problemáticas. A história de Artur Murino Mafumo, médico tradicional do sul de Moçambique, e dos objetos que lhe foram confiscados pelas autoridades coloniais na década de 1950, encontra-se bem documentada, e é um exemplo conhecido e emblemático do espólio etnográfico do IICT (Roque, 2000) (Figura 3). Mafumo, referido nos relatórios da Missão Antropológica de Moçambique como o ñanga da Matola, um “curandeiro”, foi preso pelas autoridades coloniais por se encontrar alegadamente associado a uma tentativa de homicídio e subsequentemente entrevistado, fotografado e descrito pela equipa da missão. O conjunto formado pelos seus instrumentos de trabalho, objetos sacralizados compreendidos como indissociáveis de si próprio, e dos quais dependiam as suas práticas e as suas capacidades místicas, foi-lhe confiscado após a sua detenção e depois pormenorizadamente registado (os objetos foram descritos, desenhados e fotografados) pelos investigadores portugueses. A equipa ainda traduziu, com a ajuda de um intérprete local, receitas e terapêuticas utilizadas e transmitidas por Mafumo ao longo dos três dias em que foi entrevistado na prisão. Esse grupo de objetos, classificados como etnográficos, juntamente com os restantes dados recolhidos, foram trazidos para Portugal pela equipa da missão, permanecendo Artur Murino Mafumo detido (Roque, 2000). Integra a coleção de etnografia outro conjunto de objetos com uma história com contornos em tudo semelhantes, mas menos documentada; também esses artefatos, propriedade original de um médico tradicional que foi preso, foram apreendidos pelas autoridades. E é ainda de referir um grupo de esculturas cerimoniais da Guiné-Bissau, também da coleção etnográfica, que, à semelhança dos dois exemplos anteriores, são objetos sacralizados, que se encontram associados a práticas espirituais e religiosas, e aos quais seriam reconhecidas propriedades sobrenaturais pela respectiva comunidade de origem.

Esses materiais remetem para exemplos específicos da violência epistémica exercida pelo Estado colonizador sobre saberes e práticas tradicionais, que ora se procurava registar e conhecer de forma exaustiva e global, no quadro de uma utopia colonial de conhecimento total sobre os povos e territórios subjugados (Deussen, 2022), ora eram desmerecidos e ativamente reprimidos. Mais uma vez, salientamos que esses não serão, certamente, os únicos objetos dessas coleções a terem sido apreendidos, retirados à força das suas comunidades de origem, no âmbito dessas missões. Contudo, tratam-se de casos específicos em que, excecionalmente, as próprias fontes oficiais documentam e reconhecem a repressão e a violência associada aos métodos de aquisição dos objetos. Esses exemplos tornam, desse modo, palpável a relação simbiótica e a absoluta indissociabilidade que se estabeleciam entre o poder e o controlo estatais e a ciência colonial, que prosseguiam, juntos, as suas agendas, alimentando-se e coadjuvando-se mutuamente, cada qual com os seus métodos e procedimentos próprios, sempre que necessário e possível (Cascais, Costa, 2019).

Os últimos conjuntos de objetos da coleção etnográfica que foram considerados extremamente sensíveis são constituídos, por um lado, pela coleção etnoarqueológica de Angola, maioritariamente adquiridos a um colecionador particular, e, por outro lado, os objetos que compõem a coleção etnográfica de Timor-Leste e que terão sido recolhidos pelo investigador António de Almeida, ao longo da Missão Antropológica de Timor, de onde se destaca um conjunto de estatuetas de cavalos. Em relação aos primeiros, não existem dados acerca do contexto de recolha da coleção, uma vez que se tratam de objetos comprados a colecionadores particulares ou doados pelo arqueólogo Eduardo da Cunha Serrão, que integraram as coleções do IICT entre 1978 e 1985. Quanto à segunda coleção, também se desconhecem as circunstâncias em que foi constituída, uma vez que foi incorporada nas coleções do IICT por meio de uma doação da família de António de Almeida após a sua morte, em 1984. Não existindo documentação associada, e, por conseguinte, apenas parcas informações quanto ao percurso desses objetos até à sua entrada na instituição, torna-se bastante difícil a sua contextualização histórica (Costa, Coelho, 2021, p.131, 135).

Desconhecendo-se em que moldes ocorreu a obtenção dos objetos junto das comunidades de origem, ou, no caso das coleções arqueológicas, os dados associados ao contexto do qual terão sido exumados, a avaliação e discussão ético-moral sobre a legitimidade da sua posse por parte do museu fica comprometida. O mesmo acontece com a promoção de diálogos multilaterais e o desenvolvimento de planos de ação com vista a possíveis restituições às comunidades ou países de origem, que devem, cada vez mais, ser assumidos como uma responsabilidade e um dever pelos museus que detêm património cultural associado a contextos coloniais^9^ (Förster, 2016; Grimme, 2020).

Outra coleção considerada extremamente sensível foi o conjunto de documentação textual, cartográfica e de fotografia que se convencionou designar como Arquivo de Fronteiras. Essa coleção documental foi produzida no âmbito das missões de delimitação e demarcação de fronteiras levadas a cabo nos territórios africanos e asiáticos colonizados pelo Estado português, sobretudo após a Conferência de Berlim (1884-1885) e até à década de 1940, embora exista documentação que remonta a 1856. Inclui um vasto acervo com várias tipologias de fontes, que vão desde os habituais relatórios e notas que documentam os trabalhos de campo e descrevem os territórios em questão, e todo o suporte visual associado (esboços, desenhos, mapas, fotografias), até documentação de cariz oficial e diplomático (Roque, 2015; Flores, 2021). Esse arquivo assume uma função diplomática e política importante, ao constituir uma fonte de informação incontornável, mesmo para os países independentes desde 1975, sobre as suas próprias fronteiras. Tendo-se assumido as fronteiras coloniais como inalienáveis na segunda conferência do Movimento dos Países Não Alinhados (Cairo, 1964), resolução depois consagrada no Programa de Fronteiras da União Africana (2007), o núcleo documental que no passado foi um importante instrumento da política colonial, materializando e tornando efetivo o domínio sobre os territórios, permanece hoje imprescindível para comprovar e legitimar a soberania dos estados independentes sobre os seus territórios. O caráter particularmente sensível dessa documentação é indiscutível, e a sua permanência numa instituição museológica portuguesa suscita questões éticas e políticas evidentes, na medida em que contém informação crítica para a atual gestão das fronteiras pelos respetivos países independentes (Roque, 2015), e levanta, também, questões particulares quanto à sua disponibilização e acessibilidade, por exemplo, para outros propósitos que não a diplomacia e a soberania dos estados, nomeadamente para fins de investigação.

Finalmente, a última coleção que se considerou merecer um estudo e um tratamento especialmente consciencioso foi o conjunto, já referido, de bustos com representações estereotipadas das populações africanas e asiáticas dos territórios colonizados por Portugal, que pontuam, como adornos, o Jardim Botânico Tropical. Da autoria do artista Manuel de Oliveira, essas esculturas foram encomendadas e produzidas para a Exposição do Mundo Português de 1940, cuja Seção Colonial ocupou o Jardim. A exposição, organizada em plena Segunda Guerra Mundial pelo Estado Novo, na figura do diretor do Secretariado da Propaganda Nacional, António Ferro, tinha como desígnio comemorar o duplo centenário da fundação de Portugal (1140) e da “Restauração” da independência face a Espanha (1640),^10^ tendo constituído o pináculo da propaganda nacionalista do regime, que apresentou, de forma glorificadora e laudatória, episódios e elementos da história do país. Encontrando-se a ideologia nacionalista do Estado Novo fortemente alicerçada na “mística imperial” e numa pressuposta “missão colonizadora e civilizadora de Portugal fora da Europa”, a apresentação das colónias, das suas riquezas e das suas populações assumiu-se como um elemento importante na narrativa da Seção Colonial (Castelo, 2021b, p.9-10). A sua representação reproduzia os estereótipos ocidentais da época associados aos territórios coloniais, sugerindo de forma clara uma hierarquia de culturas e civilizações em que os povos africanos, cujas manifestações culturais mereciam pouco destaque, ocupavam uma posição evidentemente inferior; já os territórios africanos, por oposição, eram celebrados pela sua abundância de recursos e potencial económico e comercial (Vargaftig, 2016). Seguindo o modelo europeu das exposições mundiais do século XIX – que persistiu na centúria seguinte –, que incluía a exibição de pessoas, a Seção Colonial incluiu um setor onde foram recriadas aldeias e contextos de habitação dos povos colonizados, em que foram instaladas, durante todo o período em que decorreu a Exposição, 138 pessoas trazidas para Lisboa de Guiné-Bissau, Angola, Moçambique, Cabo Verde, Macau, Timor-Leste, São Tomé e Príncipe e Índia, numa representação performativa das diversidades étnica e cultural do império, que suscitou o fascínio dos visitantes brancos europeus, e promoveu a objetificação humilhante e a depreciação dessas populações (Matos, 2014). Ainda que não sejam os únicos elementos materiais da Exposição de 1940 que se podem observar ainda hoje no Jardim Botânico Tropical, os bustos permanecem como uma marca evidente da ideologia racista e colonial veiculada pelo Estado Novo neste espaço.

### Contributos para uma gestão ética de patrimónios sensíveis

A caracterização do património científico-cultural do IICT, atualmente à guarda do Muhnac, o reconhecimento do seu carácter problemático e a identificação de objetos extremamente sensíveis do ponto de vista histórico e cultural constituíram um primeiro passo, essencial, para a revisão dos discursos e das narrativas que se apresentam aos públicos sobre essas coleções e para a sua reapropriação e ressignificação pelas comunidades de origem, que estiveram décadas arredadas da tutela, do controlo e da interpretação de um património que é seu. Se estruturalmente as coleções etnográficas serviram, sobretudo, para construir e comunicar ideias sobre as sociedades e as comunidades que haviam produzido e utilizado os objetos que as formam, promovendo, propositada ou inadvertidamente, ideias estereotipadas e discriminatórias sobre elas, os últimos anos introduziram alterações profundas na forma como esse tipo de património é compreendido. Vários contributos têm demonstrado o papel histórico, indiscutível, dos museus na fixação e na difusão do ideário imperialista e colonial, bem como para o domínio epistémico sobre o mundo colonizado (Mackenzie, 2009; Vergès, 2024). Coleções constituídas em contextos coloniais testemunham, frequentemente, histórias de violência e subjugação. Tornou-se, desse modo, consensual que é fundamental que as instituições atentem e procurem aferir as circunstâncias históricas em que as coleções foram constituídas e que integrem essa contextualização na forma como o património é apresentado e comunicado.

No seguimento dessa auditoria às suas coleções, o Muhnac tem procurado, nos últimos anos, melhorar e complexificar a contextualização e as narrativas históricas de exposições já existentes. Por outro lado, foi adotada como prioridade a revisão da linguagem e do vocabulário utilizados na descrição e comunicação dos objetos e coleções. Nesse sentido, partindo do exemplo do Tropenmuseum de Amsterdão (Modest, Lelijveld, 2018), está em curso a elaboração de um guião com recomendações relativamente à terminologia utilizada para descrever os objetos e os contextos históricos e realidades civilizacionais africanas e asiáticas associados, atendendo às especificidades da língua portuguesa e das coleções do museu. Pretende-se que esse documento seja, posteriormente, disponibilizado em acesso aberto no site do museu, para que possa ser utilizado por estudantes, académicos, profissionais de museus e demais pessoas interessadas. Para além disso, foi criada uma aplicação móvel para visitantes do Jardim Botânico Tropical, em que, entre outros conteúdos, é disponibilizado um percurso temático que contextualiza as épocas históricas desse espaço, com destaque para a Exposição do Mundo Português, tendo também sido colocada uma placa no jardim, com um texto que reconhece e memorializa o sofrimento, a injustiça e a humilhação provocados a pessoas de territórios colonizados pelo Estado português na recriação de “aldeias etnográficas” no contexto daquele evento.

É, ainda, de referir que está em curso um trabalho de requalificação das legendas de uma das exposições de longa duração patentes no museu, sobre o uso tradicional e local da flora por populações africanas, asiáticas e americanas, e a incorporação dessas mesmas plantas na ciência europeia a partir do império colonial português, com particular ênfase nos séculos XIX e XX. A exposição, intitulada “Plantas e povos”, está patente desde 2017, e foi a primeira exposição do Muhnac a integrar e divulgar as coleções do IICT. Esse trabalho de adaptação das legendas tem como principal objetivo atualizar a narrativa da exposição no quadro da atual reflexão em torno das coleções constituídas em contexto colonial e da linguagem utilizada para as descrever, procurando acrescentar-se novas camadas de informação, com vista a promover a reflexão dos visitantes. O caso específico dessa exposição e a atenção ao vocabulário que deve ser utilizado em exposições futuras têm revelado, por exemplo, a necessidade de promover um trabalho continuado no sentido de se identificarem e privilegiarem os nomes originais atribuídos não só a pessoas e localidades, mas também aos próprios espécimes de história natural, conforme tem vindo a ser apontado e recomendado por diversos autores (St. George, 2024; Gillman, Wright, 2020; Guedes et al., 2023).

Por outro lado, tem-se procurado desenvolver uma programação cultural crítica, socialmente inclusiva e alargada, especialmente direcionada a temas, problemáticas e questionamentos da “descolonização”, com a comemoração do Dia de África, debates públicos e encontros científicos, atividades educativas, e por meio das artes performativas e da promoção de sessões públicas de leitura, nomeadamente de manuscritos originais dos arquivos do museu. Igualmente, as exposições externas resultantes de projetos de investigação^11^ acolhidas pelo museu têm trazido à luz outro tipo de perspetivas, menos eurocêntricas, sobre a produção de conhecimento e a circulação de informação sobre os territórios colonizados (Flores, Mateus, Vasconcelos, 2024).

Contudo, o papel e a responsabilidade social dos museus não se esgotam na comunicação e na difusão das suas coleções. Reconhecer que as coleções são sensíveis e assumir a sua história colonial e a violência que lhes está associada significa, também, compreender que a gestão dessas coleções levanta problemas específicos no que respeita à sua inventariação e categorização, a nível interno, e também em relação à sua acessibilidade e disponibilização pública (Agostinho, 2019; Nordholt, Reichgelt, 2022). As coleções e o arquivo fotográfico e documental que lhes estão associados encontram-se, como a generalidade das coleções e arquivos coloniais europeus, organizados e descritos segundo uma lógica e uma estrutura que refletem o contexto e os diversos preconceitos e conceções próprias do tempo em que foram criadas, o que influencia indelevelmente a forma como se interage com esses materiais e o tipo de leituras, usos e interpretações que se pode fazer sobre eles. Assim, importa ter presente que as lógicas de organização originais das coleções reproduzem, por si só, os modos de pensar coloniais, podendo assim contribuir para perpetuar um racismo sistémico, institucional e epistemológico, e ditando a sobrevivência de ideias eurocêntricas e colonialistas. Deve, desse modo, ser assumida a necessidade de o museu estimular outras formas de gestão do seu património, desafiando essas lógicas de organização institucional que, em larga medida, ainda permanecem inalteradas. Reconhecendo-se a premência desse debate, essas são questões para as quais o museu ainda não implementou mudanças de fundo.

Por outro lado, sobretudo no que respeita à extensa coleção de fotografia, há um debate em curso que tem demonstrado os problemas éticos levantados pelo uso e pela divulgação livre de imagens produzidas em contextos de relações de poder profundamente desiguais; com a digitalização em massa e a disponibilização *online* desses acervos, as pessoas visualmente representadas nesse tipo de documentos tornam-se, uma vez mais, alvos anónimos do olhar questionador de estudiosos e observadores de todo o mundo, e as suas imagens potencialmente escrutinadas até ao mais ínfimo detalhe. Se hoje se questiona a legitimidade do colonizador ao produzir essas imagens sem qualquer atenção ao consentimento das pessoas representadas, o mesmo problema se coloca com a divulgação dessas imagens no presente (Nordholt, Reichgelt, 2022, p.72). Assim, neste momento, as imagens correspondentes aos arquivos que se mantêm *online* na plataforma Arquivo Científico Tropical Digital (criada em 2008) – e, futuramente, na plataforma que se encontra em preparação pelo museu – são, sobretudo, das missões geográficas e de história natural, ilustrando processos de trabalho de campo, onde se incluem todos os intervenientes. No entanto, as fotografias associadas às missões antropológicas, por serem, como vimos, consideradas particularmente sensíveis, permanecem apenas descritas, sem imagem publicada *online*; o acesso às imagens é realizado mediante requisição, à semelhança do que acontece com os restantes arquivos não digitalizados. É uma medida inicial, que reconhece a inevitável dualidade entre a necessidade de se garantir a proteção de dados pessoais e a consequente perda de acessibilidade, a nível global, de uma coleção que é cada vez mais requisitada. Seria benéfico que novas soluções fossem avaliadas, com base na literatura científica especializada e na experiência de outras instituições com coleções que suscitam problemas éticos semelhantes.

## Considerações finais

Neste artigo apresentou-se, de forma sistemática, o trabalho em curso de identificação e caracterização de coleções histórica e culturalmente sensíveis sob gestão do Muhnac. Procurámos descrever e explicar os critérios utilizados nessa caracterização, com base em recomendações internacionais para o tratamento desse tipo de objetos. Até ao momento, o trabalho desenvolvido centrou-se particularmente nas coleções constituídas no âmbito de missões científicas coloniais promovidas pelo Estado português nos séculos XIX e XX. O futuro e a continuidade deste trabalho passam, necessariamente, pelo aprofundamento do estudo de proveniência de todas as coleções do museu – incluindo as coleções científicas cronologicamente mais recuadas, resultantes de expedições científicas dos séculos XVIII e XIX ao Brasil e a África –, bem como pela reinterpretação, de forma crítica, do passado colonial que testemunham. É também essencial alargar este debate à sociedade civil, para que se possa acrescentar novas camadas de informação às coleções, informações essas potencialmente de caráter mais humanista e social, e que complexifiquem aquilo que se entende por conhecimento, que não se esgota no paradigma ocidental e positivista da ciência. Nesse sentido, nos últimos anos, o museu tem apoiado e acolhido iniciativas externas de entidades e grupos nacionais e internacionais ligados à academia e às artes. Consideramos que é importante aprofundar esses estudos e debates e assumir, de forma transparente, a dominação e a violência associada à criação dessas coleções, bem como a normalização dos atos coloniais em benefício da ciência. Mas outra frente de atuação necessária, e que ainda se encontra num estádio inicial, diz respeito à desobjetificação das naturezas, paisagens, pessoas e culturas representadas nessas coleções, e um esforço pela recuperação de nomes, vozes e práticas obliteradas pelo sistema de documentação. Esse esforço traduz-se, por exemplo, na recuperação e no reconhecimento da agência dos múltiplos intervenientes dessas missões, nomeadamente os trabalhadores locais cujos contributos foram, como vimos, essenciais para a produção de conhecimento no contexto da ciência colonial.

Para tal, a colaboração estreita com académicos dos países de onde provêm as coleções, bem como com representantes das comunidades de origem, é desejável, assumindo-se que deverá ser assegurada uma relação eticamente responsável, com a devida compensação dos intervenientes e total transparência relativamente à autoria dos contributos.

**Figura 1:**
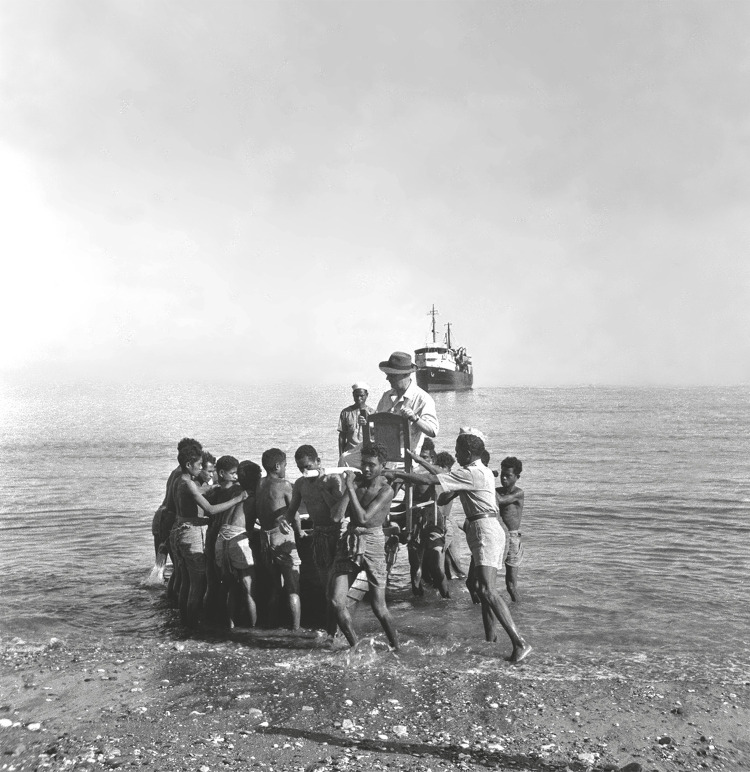
: António de Almeida, Professor de Etnologia e Etnografia Colonial na Escola Superior Colonial, no desembarque na praia Pante Macassar, em Oecussi, a ser transportado por um grupo de homens e crianças (Missão..., 1953)

## NOTAS

1 O Estado Novo foi o regime ditatorial e repressivo que vigorou em Portugal entre 1933 e 1974. Com uma matriz católica e colonialista, a sua ideologia imperial, materializada no Ato Colonial de 1930 e consagrada constitucionalmente em 1933, concebia as colónias como inalienáveis e compreendia a “missão histórica” de colonizar e civilizar como elementos inerentes e indissociáveis das próprias nacionalidade e identidade portuguesas. Essa ideologia teve largo consenso social e político no país, em torno do carácter positivo e benéfico do império e da sua legitimidade; baseou-se, em larga medida, num pressuposto de que o povo português teria uma particular vocação para se relacionar, de uma forma tolerante e benévola, com outros povos e culturas, obliterando a dimensão violenta, desigual e racista do colonialismo (Rosas, 1995; Castelo, 1999).

2 Em 1951, uma revisão constitucional substitui a designação de “Império” e “Colónias” por “Ultramar” e “Províncias Ultramarinas.” O Ministério das Colónias passa a Ministério do Ultramar e a Junta adota, também, a nova designação (Castelo, 2012b).

3 Esta publicação, cuja primeira edição é de 2018, foi sendo sucessivamente atualizada, tendo a sua terceira e última edição, que aqui se cita, sido publicada em 2021.

4 Esse critério, apesar de não ser explícito nas *Guidelines for German museums care of collections from colonial contexts* ou em documentos orientadores de outros países, foi incluído no caso das coleções científicas coloniais do Muhnac, dado que o fundamento científico para a recolha de algumas coleções foi considerado “fora de tempo” e não pertinente ou, pelo menos, bastante questionável.

5 Normalmente mencionadas, em Portugal, como Guerra Colonial ou Guerra do Ultramar. Tratam-se dos conflitos travados entre o Estado português e os movimentos independentistas de Angola, Cabo Verde, Guiné-Bissau, Moçambique e São Tomé e Príncipe entre 1961 e 1974, e que resultaram na independência das antigas colónias portuguesas em África (Cardina, 2020, 2023).

6 As primeiras fotografias datam de 1890 e vêm até ao período pós-independências, sob a designação de “missões de cooperação científica”. Das 32 coleções identificadas até ao momento, apenas três se inscrevem nesse último período.

7 A partir de 1926, às populações africanas dos territórios colonizados pelo Estado português era atribuído um estatuto político, civil e criminal de “indígenas”. No entanto, data de 1899 o Regulamento do trabalho indígena, revelando que a categoria de “indígena” já existia no enquadramento jurídico. Esses estatuto e regulamento, que conheceram várias versões até à sua abolição na década de 1960, determinavam a obrigatoriedade de as populações africanas colonizadas trabalharem. Esses instrumentos, que estabeleciam juridicamente uma diferenciação, de base racial, entre colonos e colonizados, eram fundados numa crença na inferioridade dos povos africanos, justificando e legitimando a colonização como uma “missão civilizadora” (Meneses, 2010; Neto, 2010; Jerónimo, 2010).

8
*Bertillonage* foi uma técnica criada para fins de identificação criminal em finais do século XIX pelo criminologista francês Alphonse Bertillon.

9 Este dever encontra-se, aliás, consagrado no Código de Ética do Icom (Icom, 2017).

10 Na historiografia portuguesa, designa-se como “Restauração” o término do período de 60 anos (1580-1640) em que, fruto de uma crise de sucessão em Portugal, houve uma união dinástica das coroas portuguesa e espanhola, sob a dinastia Habsburgo.

11 Como exemplo, as exposições “Impulso fotográfico. (Des) Arrumar o arquivo colonial” ou “Panorama do Congo. Desenrolar o passado com a realidade virtual”.
